# Access to information and decision making on teenage pregnancy prevention by females in Tshwane

**DOI:** 10.4102/curationis.v38i2.1540

**Published:** 2015-11-05

**Authors:** J.P.F. Masemola-Yende, Sanah M. Mataboge

**Affiliations:** 1 Department of Nursing Science, University of Pretoria, South Africa

## Abstract

**Background:**

The increase in the number of teenage pregnancies and its negative consequences has encouraged various researchers to explore the possible causes of teenage pregnancy. Findings from previously-conducted research have indicated different preventable factors that predispose female teenagers to pregnancy, such as staff attitudes and the lack of information resulting from poor access to health facilities.

**Objective:**

To explore and describe access to information and decision making on teenage pregnancy prevention by females using a primary healthcare clinic in Tshwane, South Africa.

**Method:**

In this study, the researchers used a descriptive qualitative and exploratory research design to explore and describe the verbal reports regarding prevention of teenage pregnancy by females using a primary healthcare clinic in Tshwane, South Africa. Face-to-face semi-structured interviews were conducted with 15 female participants aged between 15 and 26, who had been pregnant once or more during their teens.

**Results:**

Two themes emerged, namely, access to information and decision making by female teenagers. Five categories that emerged were: access to information on pregnancy prevention; ignoring of provided information; the use of alternative medicine with hormonal contraception; personal reasons for use and non-use of contraception; and decisions made by teenagers to not fall pregnant. Females in this study fell pregnant in their teens, even though they had access to information.

**Conclusion:**

Given the complexity of this problem, female teenagers should use their families as primary sources of information for reproductive health promotion and educational institutions should build on this to aid the prevention of teenage pregnancy.

## Introduction

Teenage pregnancy is a global reproductive health promotion problem that affects female teenagers, families and communities, both in developed and developing countries, as children aged 10 to 19 years, unmarried and still at school, become pregnant (Mchunu et  al. 2012:428). According to Marino et  al. (2013:1029), teenage pregnancy may occur as early as age 10 to 12, prior to the teenage years. Occurrence of pregnancy prior to the teenage years is related to age at menarche, which is commonly used as a milestone for pubertal development and the onset of reproductive capability. Early age of menarche, such as nine to 10 years, predisposes and is related to early sexual debut and unintended pregnancy amongst female teenagers (Marino et  al. 2013:1029). Teenage pregnancy may occur at puberty because ovulation can occur before the first menstrual period. It is thus important for teenagers to acquire appropriate knowledge and an understanding of their body and its functions as part of their life skills so that they are able to prevent pregnancy. Mataboge, Beukes and Nolte (2014:135) and Willan (2013:4) assert that lack of knowledge and misconceptions on conception expose female teenagers to unintended pregnancy.

Basic knowledge possessed by females on the physiology of the menstruation cycle and conception is known as functional health literacy. Functional health literacy is a domain of health literacy which Manganello (2008:840) defines as ‘the degree to which individuals have the capacity to obtain, process, and understand basic health information and access services needed to make appropriate health decisions’. Ishikawa and Yano (2008) define functional health literacy as the use of basic information and understanding the importance of self-care. Functional health literacy is one of three categories of health literacy classified by Nutbeam (as cited in Kickbusch 2001:292), that would promote self-care – including the prevention of unsafe sexual practices. The second health literacy category is communicative health literacy, which includes identifying the best sources of information, an ability to seek personally-adapted information from a medical team and the application of knowledge on a daily basis. The third health literacy category is critical health literacy, which is said to involve more advanced cognitive skills. These cognitive skills, together with social skills, can be applied to analyse information critically and to use this to exert greater control over life events and situations. The last two categories require teenagers to understand the determinants of health and negotiate any modification thereof. Some teenagers seem to be unable to negotiate modification of determinants of reproductive health, hence teenage pregnancy continues to be a problem. Low involvement or absence of involvement of female teenagers in decision making regarding the use of contraception or condoms also contributed to early pregnancy (Acharya et  al. 2010:5).

Mangiaterra et  al. (2008:2) reported that a total of 16 million female teenagers aged 15 to 19 give birth each year worldwide. This accounts for 11% of all births, with 95% of these births occurring in developing countries (Mangiaterra et  al. 2008:2). In South Africa, a total of 160 754 teenage pregnancies were recorded between 2010 and 2011, as stated by Statistics South Africa and the Department of Basic Education (cited by Mpofu 2012:1). A study by Grant and Hallman (2008:369) reported that one in five 18-year-old females in South Africa had given birth, with more than 40% becoming mothers by the age of 20. Teenage pregnancy may be coupled with complications associated with a variety of obstetric, social, educational and health-related problems, hence it is important to prevent its occurrence (Collier 2009:393).

The high rate of teenage pregnancy may be attributed to sexuality education in schools that is not mandatory as some faith-based schools are not providing it as part of their curriculum. Females who are not provided with information on the risk of teenage pregnancy may present with low functional health literacy, which could expose them to teenage pregnancy (Adeokun et  al. 2009:38). A study conducted by Ishikawa and Yano (2008:117) and Mataboge et  al. (2014:136) acknowledged that limited information and a lack of functional health literacy have a negative impact on decision-making skills and health outcomes. Furthermore, limited access to information is associated with less knowledge on the use of contraception services and pregnancy prevention. Decision-making skills are part of the functional health literacy that female teenagers need to possess regarding the use of contraception. A study by Ziyane and Ehlers (2007:10) into the functional health literacy of females with regard to pregnancy prevention found that female teenagers possessed insufficient or no information about contraception, which could negatively affect decision making regarding pregnancy prevention. Meanwhile, Willan (2013:7) reiterates the fact that as teenage pregnancy is related to multiple factors, including health literacy, the response needs to address all factors from the social and the structural through to individual behaviours, all of which can empower teenagers to make informed choices and decisions about their sexuality. Access to information which will enhance the level of female teenagers’ functional health literacy on the prevention of pregnancy may curtail the observed rates of pregnancy amongst teenagers.

In order to enhance the prevention of teenage pregnancy and development of health literacy in South Africa, loveLife, a non-governmental organisation, was established to facilitate the development of health literacy on reproductive and sexual health services for teenagers (Peltzer & Chirinda 2014:77, 79). However, the problem of teenage pregnancy persists, especially amongst females whose self-esteem and expectations are low, as well as those living in poverty and in areas with high unemployment and few work opportunities (Braine 2009:411). Involvement of parents to ensure that teenagers obtain access to information on pregnancy prevention may mitigate the factors that predispose female teenagers to pregnancy.

Society in general must accept teenagers as sexual beings and should contribute to the prevention of teenage pregnancy, by providing females with information and decision-making skills, whether they are sexually active or not. Teenage pregnancy is considered to be, in part, the result of a lack of knowledge about contraception and human physiology (Ehlers 2003:15). This study explored the verbal reports by female teenagers on access to information and decision making regarding teenage pregnancy prevention, in a primary healthcare clinic in Tshwane, South Africa.

### Problem statement

The incidence of teenage pregnancies amongst learners of school-going age was reported to be 20 000 and primary school learners accounted for 223 of the total number of pregnancies amongst learners in South Africa in 2014 (South African Broadcasting Company [SABC] NEWS 2015). In Gauteng Province, South Africa, a 13-year-old at a technical secondary school in a township gave birth to a baby boy in June 2010 (Govender 2010:6). Teenage pregnancies in under-16-year-olds in all provinces were estimated at 12 120 – just over a quarter of the total of 45 276 learners that fell pregnant in 2009. Gauteng recorded the highest number, namely, 109 pregnancies, amongst learners in primary schools, with 74 being Grade 3 learners (SABC NEWS 2013). Teenage pregnancy remains a health problem, even though information on prevention is provided by programmes for adolescents (such as the National Adolescent-Friendly Clinic Initiative and loveLife) and at school through life orientation as a subject (Bharat & Mahendra 2007:93).

The life orientation subject provides information on menstruation and pregnancy prevention aspects, such as contraception (DoH & Department of Basic Education [DBE] 2012:12). However, some of the females in the primary healthcare setting where the researcher is employed experienced more than one pregnancy, even though they reported that they had access to information on contraception. Nevertheless, they made decisions that led to them falling pregnant. The researcher provided care to a number of 14-year-old females in ante-natal care who were attending a school where life orientation was provided as a subject, but who still did not use contraception. This motivated the researcher to conduct the study in a primary healthcare clinic in Tshwane District, South Africa in order to explore and describe the verbal reports on access to information and decision making regarding teenage pregnancy prevention by female teenagers.

### Research objectives

The objectives of the study were to explore and describe:

- access to information on teenage pregnancy prevention by female teenagers using a primary healthcare clinic in Tshwane
- decision making on teenage pregnancy prevention by female teenagers using a primary healthcare clinic in Tshwane.

#### Significance of the study

It is predicted that the findings of this study will inform healthcare providers on the information that teenagers possess on the prevention of pregnancy and what informs their decision making to ensure prevention. This may inform healthcare providers as to what health education to provide to teenagers on negotiation and decision making so as to make sure that they do not experience teenage pregnancies. Programme providers may be made aware of the importance of exploring the functional health literacy information possessed by teenagers and the decisions made by teenagers regarding prevention of teenage pregnancy.

## Research design

### Research approach and method

In this study, the researcher used a descriptive qualitative research design to both explore and describe the verbal reports provided on access to information and decision making by female teenagers at a primary healthcare clinic in Tshwane District, South Africa regarding prevention of pregnancy. The design was chosen as female teenagers and those who experienced teenage pregnancy could narrate the phenomenon as they experienced it.

#### Population and sampling

The research setting was a primary healthcare clinic located in the western urban area of Tshwane, Gauteng, South Africa, populated by low- and middle-income families. This primary healthcare clinic provides comprehensive health services 40 hours per week. Female teenagers and those who experience teenage pregnancy report at the clinic for ante-natal care, family planning and the well-baby clinic.

In this study, the population was female teenagers who were pregnant or those who had experienced teenage pregnancy and were using a primary healthcare clinic in Tshwane District, South Africa. The clinic provides bi-weekly care to an estimated total of 100 teenagers who are pregnant or are teenage mothers. For participation in the study, the researcher identified teenagers who were aged 15 to 19 years, as well as females who had been pregnant once or more during their teenage years, but were less than 26 years old. Participants were recruited from ante-natal care, the well-baby clinic, minor ailments or family planning by explaining the objectives and benefits of the study to females above 20 years of age and asking for voluntary participation. The researcher used purposive sampling to consciously select 15 participants who met the inclusion criteria, namely: pregnant teenagers; teenage mothers; and females who were no longer teenagers but had experienced teenage pregnancy. There were two participants who had been pregnant more than once during their teenage years, with the second pregnancy being at 18 and 19 years, respectively. The phenomenon explored was the access to information and decisions made by female teenagers who fell pregnant. The age of participants was therefore not restricted to the teenage years. An appointment was secured with each participant during the next visit or the earliest convenient time to be interviewed by a research assistant assigned to do the data collection. Females 18 years and older signed informed consent, whilst females under 18 years of age who were accompanied by parents during ante-natal care were included after obtaining informed consent from their parents.

The participants who were above 19 years of age were included as some had been pregnant once or more during their teenage years. Their experience on the phenomenon was needed so that they could provide verbal reports on how access to information regarding pregnancy prevention was when they were teenagers. The participants were recruited by word of mouth when they visited the clinic for mother and child services.

#### Data collection

Semi-structured interviews were used to get in depth information on the phenomenon studied (Streubert & Carpenter 2007:381). In order to avoid bias as a result of her personal experience, the researcher appointed the research assistant for data collection so as to ensure credibility and dependability. A consultation room with minimal distraction was used and interviews were audiotaped. Those pregnant teenagers accompanied by mothers were interviewed alone. In order to allay anxiety, the explanation of the interview process preceded the initiation of the interview. The same research questions were asked and probing was used. Interview sessions lasted 30 minutes and data saturation was reached with the 13th participant, after which two further participants were added to confirm data saturation. The researcher wrote the field notes and operated the audiotape recorder. Each interview and its field notes were allocated a number to represent the participant. The same research questions were asked:

- How is the access to information by female teenagers on teenage pregnancy prevention?
- How do female teenagers make decisions regarding prevention of teenage pregnancy?

#### Data treatment

Tesch’s (1990) open coding method was used (as described by Polit & Beck 2012:509). Data were transcribed verbatim and the transcripts were read repeatedly to ascertain and confirm the meaning. The main stories were identified and were written on the side so as to capture any interesting data. Those verbal reports with the same meaning were highlighted. Verbal reports with common meanings were grouped together and later converged to represent themes. Later, the statements were reduced based on the verbatim reports and were given titles to represent categories. An external coder was used to conduct data analysis and consensus was reached between the researcher and the coder regarding the titles of themes and categories.

## Results

### Participants’ demographic data

The participants’ ages ranged between 15 and 26 years. A total of 10 participants were pregnant, one 15-year-old was a teenage mother and four participants were mothers above 19 years who had babies as teenagers. Amongst the last group, two participants had experienced pregnancy twice during their teenage years. The level of education ranged from Grade 6 to tertiary. Most of the participants were single and unemployed: single (*n*= 10), married (*n*= 2), engaged (*n*= 3), unemployed (*n*= 11) and employed (*n*= 4). Some participants stayed with their parents (*n*= 10), others were married and stayed with their partners (*n*= 2), whilst some were cohabitating (*n*= 2) and one was single and lived alone (*n*= 1).

Two themes emerged: access to information and decision making regarding the use of contraception. During the discussion of results, verbatim quotations were used to support the categories and provide evidence.

### Theme one: Access to information

An enabling environment has been created to mitigate the occurrence of teenage pregnancy in South Africa. School programmes, the family environment and media all support and encourage the communication of messages and sharing of information regarding the prevention of teenage pregnancy. Three categories emerged from this theme and are discussed below in more detail.

#### Access to information on prevention of pregnancy

Multiple providers of information regarding teenage pregnancy prevention were identified in this study, namely, the DBE, healthcare providers, mothers, peers, boyfriends and the media. Mothers, peers and boyfriends mainly provided information to the females with regard to accessing contraception. Life Orientation and school health programmes are provided in schools in most developing countries, primarily to educate young people about sexuality and sexual relationships, including prevention of teenage pregnancy. The school is regarded as a captive setting in which to reach a large audience of young people, hopefully before they initiate sexual activity.

Some participants reported access to the provision of information even though they were teenage mothers. They verbalised that:

‘They teach us at school about life skills, reproductive health, coping skills and sex education, like prevention, STI [*sexually-transmitted infections*] and HIV.’ (18 years old)

‘The school health nurses come to our school to teach us about things like menstruation, pregnancy, diseases and prevention and correct skills on condom use.’ (18 years old)

‘There is the sister that comes at school every Wednesday to teach us about condoms, HIV and STI.’ (15 years old)

Access to information regarding pregnancy prevention services was provided by mothers, especially those who became concerned when discovering that their teenage daughters were having sexual relationships with boys. The first thing that came to the mind of those mothers was that their daughters were now sexually active and this would lead to pregnancy, STI and or HIV. Mothers gave information as reported by the participants:

‘When my mother saw me with a boy for the first time, she said “sho, sho, you must go to the clinic for injection.”’ (24 years old)

‘My mother explained to me that “my child I see that you are now in love. You must go to the clinic and inject because when you are going to sleep with that person, you are going to be pregnant.”’ (26 years old)

‘I told my mother that I am going to stay with my boyfriend and she said it again that I am going to fall pregnant. In February 2006 I left school and came to Pretoria to stay with my boyfriend. When I came to Pretoria I forgot about prevention and my mother's words. In April I fell pregnant for the second time. In July my mother recognised that I am really pregnant and I said that I have only gained weight as I have a child.’ (24 years old)

In this study, peers included friends and boyfriends and were reported as providers of information because females discuss many topics freely with their peers which they would rarely discuss openly with their mothers, sisters or healthcare providers. Some female teenagers were geared toward teenage pregnancy prevention and shared such information with their peers. Friends and boyfriends were reported to be providers of information as follows:

‘I used to talk with my friends at school about going to the clinic for prevention, boyfriends and having sex.’ (16 years old)

‘My boyfriend once told me that I must go to the clinic for injection.’ (18 years old)

The media is also a source of knowledge and information on contraception in this study, as reported by participants. The participants viewed the media as a source of information, especially for issues such as sexual health and sexual intercourse. Participants reported that:

‘On the radio there is a sister who talks about prevention and diseases; even in *True Love* magazine you can read about it.’ (18 years old)

‘TV stories like “Intersections” talk about sex [*sexual intercourse*] and use of condoms.’ (19 years old)

#### Ignoring the provided information

On the other hand, it was observed in this study that opportunities afforded to female teenagers to develop health literacy were ignored and were sometimes deliberately undermined by these females, who then fell pregnant. One participant who had a baby at the age of 16 said:

‘I was not listening in class because I don’t like topics on pregnancy prevention, and Life Orientation class is the one I was not attending, I was going out when it starts.’ (16 years old)

Another one said:

‘I did know but I would not listen to nobody. I was drinking and mixing with the wrong friends.’ (17 years old)

Knowing how pregnancy occurs did not help to prevent a second teenage pregnancy, because the experience was ignored, as described in the following excerpt:

‘I had the miscarriage three years back at the age of 15 years. I did not know that I am pregnant, I missed menstruation for three months then suddenly saw a lot of blood. I went to the hospital and I was told that it is a miscarriage and was told to use an injection. I stopped it and now I am 18 years and pregnant again.’ (24 years old)

#### The use of alternative medicine with hormonal contraception

Some of participants used alternative medicine as they possessed inaccurate information even though they accessed contraception services. Participants who used hormonal contraception reported risky behaviour that would interfere with the effectiveness of the method. Participants in this study thought that herbs are the solution for managing side-effects such as amenorrhoea, which is encountered whilst using hormonal contraception. This was expressed as follows:

‘If you know that you are going for injection, two days before you can use *izimbiza* [*herbal concoction*] like to clean, to clean yourself from the previous injection, to prepare yourself for the fresh one.’ (26 years old)

Another participant stated that:

‘When you use injection you don’t menstruate, so you need to go to the traditional doctor to *go phatsa* [*get incisions*] so that the blood which has accumulated inside you comes out, otherwise you will get sick.’ (18 years old)

### Theme two: Decision making regarding the use of contraception

The theme on decision making regarding the use of contraception emerged with two categories, which are discussed in more detail below.

#### Personal reasons for use and non-use of contraception

Central to teenage pregnancy prevention in this study was the decision-making skills that acted as an enabler for the females who are at risk of teenage pregnancy. A participant who experienced teenage pregnancy twice, the first pregnancy being at 16 years of age and the second at 18, reported fear of a needle as a leading factor for non-use of contraceptive injections. The participants knew about contraception; however, they had personal reasons not to use contraception. Some female teenagers were not mentally prepared to use either injections or pills or to comply with the method. Condom use was also not constant as the participants reported problems of size and holes in the condom. Another participant who had been pregnant twice by the age of 18 said:

‘I was scared of the injection, the pills I was going to forget them. I was scared of the needle and after my first child I prevented with Nur-isterate for only one month.’ (24 years old)

‘I heard some sisters saying that the injection makes your body to swell and shiny [*sic*].’ (18 years old)

Possession of knowledge did not facilitate compliance with oral hormonal contraception as verbalised by the participant in the following excerpt:

‘I used to drink pills a week before sleeping with my boyfriend because he is staying in Johannesburg and I don’t have a boyfriend this side. It is boring to drink them when you know that you are not going to have sex with anyone.’ (19 years old)

Another participant said:

‘Pills makes your body to build up [*sic*] thus I stopped taking them.’ (18 years old)

To some teenagers, the side effects of hormonal contraception, specifically the injectable contraception, were enablers for their use. Although contraception does have side effects, some teenagers reported feeling comfortable with the side effects whilst some had mental peace as they only used the injection and they were protected from pregnancy. One said:

‘With the injection you don’t worry about buying pads [*sanitary towels*] as you don’t menstruate.’ (23 years old)

The participant further elaborated:

‘Injection makes you to gain weight [*sic*], but you forget about taking it every day, like pills which you forget easily and the next minute you are pregnant.’ (23 years old)

Even though condoms are freely accessible in the clinics, there were various personal reasons for non-use. A participant said:

‘When you put on a condom you find that it is too small and when you force to put it on it tears.’ (17 years old)

Another said:

‘Sometimes condoms have holes and tear easily.’ (16 years old)

#### Decision made by teenagers regarding falling pregnant

Decision to fall pregnant: When females are in a sexual relationship they make decisions, such as the decision to have a baby. The desire by both male and female teenagers to do so had resulted in teenage pregnancy amongst some of the females in this study. These barriers were expressed as follows:

‘We planned with the child's father that we want to have a child first before he marries me.’ (18 years old)

Another participant who experienced a third teenage pregnancy said:

‘In March 2007, I went to the clinic as I had burning urine; my urine was tested and I was told that I am pregnant for the third time. I cried as the Doctor told me that my pregnancy is 12 weeks and advised me to do an abortion and I told him that I am scared that I will die if I do the abortion. My boyfriend was very happy because our first child with him has died and I became pregnant again.’ (24 years old)

The participant explained how the decision for the third pregnancy in 2010 was made. She said:

‘I prevented [*sic*] from 2007–2010 when my boyfriend tricked me and said that I must leave prevention [*contraception*] because our baby is getting old and he is going to marry me.’ (24 years old)

Another participant knew that unprotected sex might lead to pregnancy but did not mind falling pregnant because her boyfriend promised to take her to computer school. The participant said:

‘My new boyfriend sent me money to come to Pretoria, telling me that he is going to take me to the computer school for computer lessons and will give me money. I could not resist when he did not want to use condom and I fell pregnant.’ (23 years old)

**Decisions to not fall pregnant:** Some females made the decision to use and comply with contraception only after experiencing teenage pregnancy. The decision to prevent repeated pregnancy is communicated in the following excerpt:

‘Since the sister has explained to me how the prevention works, I am now going to use injection not to fall pregnant again as marriage is scarce sister.’ (16 years old)

Another one said:

‘I will now use injection and condoms because my mother will kill me if I fall pregnant again.’ (17 years old)

Some participants used the clinics and reported that they will prevent pregnancy as they now understand how to take the contraception. They said:

‘I will not be pregnant again, I asked them [*sisters*] at the clinic [*about compliance on contraception*]. The sister that attended to me yesterday said that pills should be taken at the same time every day. Like if you drink them at six, you must drink them at six.’ (19 years old)

She further added:

‘The sister told us that if you know that you are going to have sex, you must protect yourself with a condom.’ (19 years old)

## Ethical considerations

Ethical permission was obtained from the university's research ethical committee and the manager of the primary healthcare clinic. In this study, the three main ethical guidelines of the Belmont Report of 1979, as cited in Polit and Beck (2012:252) namely, beneficence, respect for human dignity and justice were used. This included the principle of respect for persons or autonomy, whereby participants are treated as autonomous and capable of making deliberate decisions about whether to participate in research (Lobiondo-Wood 2010:252; Polit & Beck 2012:152–154). Assent and consent were obtained and no participants were coerced to take part. Participation was voluntary and no names of participants were revealed; only numbers were assigned to each interview recorded and the transcripts so as to ensure that no data will be linked to a participant when reported. This was done to ensure beneficence, respect and confidentiality. A clinic social worker and psychologist located in the nearby clinic were consulted to await referral of participants who might present with emotional manifestations during the interviews. However, none of the participants were referred.

## Trustworthiness

The framework and strategies described by Lincoln and Guba (1985) (cited in Polit & Beck 2010:492) were employed to ensure the trustworthiness of data collection and interpretation thereof. Trustworthiness was ensured using the criteria for credibility, transferability, dependability and conformability. Credibility was ensured as the researcher was involved during data collection by writing field notes, conducted data analysis and data discussion as such she was immersed in the setting in order to ensure prolonged engagement. She also worked as a healthcare provider at the primary healthcare clinic. In addition, member checking was implemented in order to ascertain the credibility of the transcribed data. The research assistant was used to ascertain the dependability of the results as the research assistant bracketed by not influencing the data collection due to her knowledge of the phenomenon as she was a professional nurse during data collection. The researcher provided a thick description of the research design, methods and data discussions to ensure transferability. An audit trail of all the research processes is available to ensure conformability.

## Discussion

Evidence from the verbal reports by female teenagers in this study affirmed access to information on teenage pregnancy prevention. One of the providers of information on teenage pregnancy was described to be the DBE, who provided life orientation from Grade 4 up to Grade 12 which includes information on contraception and menstruation (DoH & DBE 2012:12). Family planning is one of the focus areas of ‘Healthy People 2010’, one of the goals of which is prevention of unintended pregnancy (Holcomb, Williams & Skipper 2009:20).

The verbal reports affirm that females grow up in an environment where information on teenage pregnancy prevention is accessible. Even though sexual health education is provided in schools in South Africa, the programme is reportedly not reaching the desired outcomes (DBE 2009:60). Holcomb et  al. (2009:20) state that a lack of relevant information available to females may hinder the teenagers in making mature sexual decisions to prevent teenage pregnancy. Female teenagers need to access information before they consider using contraceptives, emergency contraceptives or termination of pregnancy services (Ehlers 2003:15). The females in this study have access to information from different people who were concerned about their sexual health, however they still fell pregnant.

Prior to entering formal school, families are primary institutions to ensure that children acquire reproductive health promotion information and skills. It is not a new finding in this study that, although teenage females are in possession of basic knowledge on sexual health, very few of them have used the knowledge into practice to prevent pregnancy. Willan (2013:4–7) reported that access to information is in place, however teenagers do not use it in their sexual encounters. Parents in South Africa are encouraged to talk about sex and reproductive health issues by a slogan: ‘love them enough to talk about sex’ as advertised in the media (SABC advertisement 2010–2011). As a primary socialising agent of children, parents are a trusted and a primary source of information about sexuality for young people (DBE 2009:62). Mothers in this study fulfilled the responsibility to advise their daughters on pregnancy prevention, however not all daughters implemented the advice to access contraception.

Peers may also be providers of reproductive health promotion information. In this study, teenagers talked to each other regarding pregnancy prevention. Teenagers are marked by significant psychosocial transformation and recognition in their social life, spending more time with peers and socialising with a larger, more diversified cohort with strong desires to be acceptable to both their close friends and larger cohort groups (Mason et  al. 2013:1236). Programmes such as the National Adolescent-Friendly Clinic Initiative are designed to improve the quality of adolescent reproductive and sexual ‘health services at primary care level and strengthen the public sector's ability to respond to adolescent health needs’ (Dickson-Tetteh & Foy 2001, as cited in Baloyi 2009:3). In this study, information on anatomy and physiology of the reproductive system that facilitates the prevention of teenage pregnancy was provided at schools, however teenagers who were absent in these classes missed on this information. One teenager mother reported that she walked out of life orientation lessons, which may have had a negative effect.

It was evident from the results that, in addition to mothers and peers as platforms for information sharing on pregnancy prevention, public platform and media also played a part in information dissemination. Multimedia campaigns such as loveLife, Soul City and Khomanani have played a seminal role in improving young people's knowledge about sexual behaviour and, in particular, regarding HIV (DBE 2009:62). The media presents information during talk shows, short story series and internet websites. Xu, Che and Cheng (2005) (cited by Wu 2010:405) reveal that the main sources of knowledge regarding sexuality and contraception are the media, internet and peers. Ramkissoon et  al. (2010:38) report that a survey on access to information revealed that the provision of communication programmes have an impact on young people's knowledge levels and behaviour in relation to condom usage. The findings were similar in this study, as these sources of information were reported to be playing a part in functional health literacy development amongst teenagers.

The use of certain medicines, such as anti-tuberculosis drugs, reduces the effectiveness of contraception. In the case of the use of drugs that interact with the contraceptive injections, such as tuberculosis drugs, the date to repeat the injection is set two weeks earlier for norethisterone enanthate and four weeks earlier for depot medroxyprogesterone acetate. (DoH 2009:55). The participants who used herbs (*izimbiza*) whilst on injectables were at risk of drug interaction and low contraception efficacy. The UK Government (2014) reported that herbal medication lowers the efficacy of hormonal contraception and was reported to have caused unplanned pregnancy and breakthrough bleeding amongst users of St John's wort, a herbal medication used for mood treatment in United Kingdom. Clients are routinely advised to avoid the use of any drugs whilst on hormonal contraception as this may lower the contraceptive's efficacy (DoH 2012:24). Knowledge on drug interaction needs to be provided to teenagers to ensure pregnancy prevention.

The rate of teenage pregnancy remains unacceptably high, even though it is reported to have declined nationally. A high level of knowledge about modern methods of contraception is observed, however a large cohort of young people do not use contraception, whilst some use contraception inconsistently and incorrectly (DBE 2009:6). In the South African government's primary healthcare settings there are two available contraception injections, namely, depot medroxyprogesterone acetate (used three-monthly) and the two-monthly norethisterone enanthate (DoH 2012:28). Barden-O’ Fallon et  al. (2009:475) confirm that a main reason for contraceptive discontinuation is the experience of side effects, especially amongst users of hormonal methods. Weight gain with use of injections is caused by an increase in appetite if one does not manage one's eating habits or engage in physical exercise. High levels of oestrogen cause weight gain because of increased appetite and fluid retention (Freedman 2013:1). In contrast, Holcomb et  al. (2009:22) state that there is no evidence of weight gain when using combined oral contraceptives. The non-use of hormonal contraception because of the side-effect of weight gain, as mentioned in this study, may contribute to the sustained incidence of teenage pregnancy. Such misinformation needs to be corrected so as to increase the use of hormonal contraception by teenagers and improve compliance to prevent pregnancy.

The fear of needles is a personal reason rather than a side effect, however it caused one participant to fall pregnant twice by the age of 18. Her personal reasons led to teenage pregnancy however that changed after the second pregnancy when she decided to use contraception. The DoH (DoH 2012:28) has listed amenorrhoea as having health benefits to females, including prevention of iron deficiency anaemia. The injections are regarded as improving compliance, as the clients need to comply with return dates only once in two to three months. In this study, participants regarded this as an additional personal benefit. Some of the participants in this study did not have misconceptions regarding amenorrhea and did not discontinue the injection because they feared that menstrual blood was building up in their bodies (DoH 2012:31). The study results concluded that counselling about side effects was an effective way of increasing continuation rates of contraception use and acceptance of side effects.

Some teenagers made a conscious decision to fall pregnant, as was reported by those in this study who had promises of economic gain and/or marriage from their boyfriends. Teenage pregnancy is not always unintended or unplanned, as alluded to by participants in this study. In some instances, a desire to access education becomes a risk factor that exposes teenagers to falling pregnant in order to gain financial favours, either within or outside marriage. Teenagers take decisions that disregard the risk of pregnancy and STIs, including HIV, because of limited access to financial resources. Both females in this study who fell pregnant as a step to ensuring marriage and/or financial favours to complete their education did not have these promises fulfilled. The studies by Nelson et  al. (2011:277) and Maja (2007:72) confirm that mothers who are teenagers did not insist on or negotiate contraception and condom use with their steady boyfriends or ‘baby daddy’, as they trusted and loved them. Continual lack of financial resources acted as a facilitator to teenagers falling pregnant, as was reported by participants in this study. However, some teenagers are determined to prevent pregnancy. These multiple risks to unplanned teenage pregnancy point out the need for initiatives to ensure teenagers’ access to information and multi-pronged programmes to prevent teenage pregnancy (Willian 2013:7).

### Practical implications of the study

The study gave rise to various implications, as are discussed below.

#### Nursing education

Nursing curricula need to continuously update sexual, reproductive health and family planning information in order to empower healthcare providers with a broad knowledge regarding these aspects. The DoH and DBE need to review policies, curriculum development and teaching methods pertaining to teenagers’ sexual and reproductive health education.

The collaboration between education and health should strengthen programmes such as peer education through involvement of both females and boys in teenage pregnancy prevention. Continuation of health education on prevention of teenage pregnancy needs to be a joint effort whereby education is also given to mothers and other family members.

#### Nursing practice

Health policies and standard procedures need to be emphasised, as the attitudes of healthcare providers were identified as a barrier to accessibility of reproductive health services by teenagers. Respect for teenagers needs to be instilled in healthcare providers so as to improve the quality of care and to allow reproductive services to be utilised effectively.

#### Nursing research

Although much research has been conducted on teenage pregnancy, other approaches, such as teenage-centred research, need to be conducted so as to explore the factors that affect the use of and compliance with contraceptive regimes.

## Limitations of the study

The study was small, limited to pregnant teenagers and mothers out of their teens, reporting at one clinic, who had previously experienced a teenage pregnancy. It was conducted in a subdistrict, so the opportunity to generalise the findings is limited. Further research could be carried out in both the district and province.

## Recommendations

Based on the research findings and their discussions, the researcher recommend that education on reproductive and sexual health should be provided before female teenagers become sexually active, with the involvement of both male and female teenagers. All healthcare providers should be trained in contraception and adolescent care in order to motivate them to use and comply on contraception. Furthermore, multimedia campaigns such as radio talk shows and debates in the schools and community on condoms and contraception methods should be conducted in formal and informal teenagers’ groups at church and during sport activities. This will allow them to make informed choices on reproductive health issues that affect their lives.

## Conclusion

It is concluded that female teenagers have access to information but lack the skills to implement it in order to prevent teenage pregnancy. Furthermore parents, teachers and boyfriends also provide information which female teenagers decide not to use. There is thus a need to support female teenagers, either with regard to postponing their sexual debut or with regard to use of contraceptives.

## References

[CIT0001] Acharya D.R., Bhattarai R., Poobalan A., van Teijlingen E.R. & Chapman G., 2010, ‘Factors associated with teenage pregnancy in South Asia: A systematic review’, *Health Science Journal* 4(1), 3–14.

[CIT0002] Adeokun L.A., Ricketts O.L., Ajuwon A.J., & Ladipo O.A., 2009, ‘Sexual and reproductive health knowledge, behaviour and education needs of in-school adolescents in northern Nigeria’, *African Journal of Reproductive Health* 13(4), 37–49. PMID: 20690272. 20690272

[CIT0003] Baloyi G., 2009, *The evaluation of national adolescent-friendly clinic initiative (NAFCI) programme in greater tzaneen sub-district, Limpopo Province, South Africa*, Masters’ thesis, Health Studies, University of South Africa.

[CIT0004] Barden-O’Fallon J., Speizer I., Rodriguez F. & Calix J., 2009, ‘Experience with side effects among users of injectables, the IUD, and oral contraceptive pills in four urban areas of Honduras’, *Health Care for Woman International* 30(6), 475–483. PMID: 19418321, 10.1080/07399330902801187 19418321

[CIT0005] Bharat S. & Mahendra V.S., 2007, ‘Meeting the sexual and reproductive health needs of people living with HIV: Challenges for health care providers’, *Reproductive Health Matters* 15(29), 93–112. PMID: 17531750, 10.1016/S0968-8080(07)29030-5 17531750

[CIT0006] Braine T., 2009, ‘Adolescent pregnancy: A culturally complex issue’, *Bulletin of World Health Organization* 87(6), 405–484. PMID: 19565115. 10.2471/BLT.09.020609 PMC2686202 19565115

[CIT0007] Collier J., 2009, ‘The rising proportion of repeat teenage pregnancies in young women presenting for termination of pregnancy from 1991 to 2007’, *Contraception* 79(5), 393–396. PMID: 19341853, 10.1016/j.contraception.2008.11.014 19341853

[CIT0008] Department of Basic Education, 2009, *Teenage pregnancy in South Africa: With specific focus on school-going learners*, Government Printers, Pretoria.

[CIT0009] Department of Health, 2009, *National tuberculosis management guidelines 2009*, Government Printers, Pretoria.

[CIT0010] Department of Health, 2012, *National contraception clinical guidelines: A companion to the national contraception and fertility planning policy and service delivery guidelines*, Government Printers, Pretoria.

[CIT0011] Departments of Health and Basic Education, 2012, *Integrated school health policy**,* Government Printers, Pretoria.

[CIT0012] Dickson-Tetteh K. & Foy D., 2001, *The national adolescent-friendly clinic initiative: Handbook of adolescent sexual and reproductive health care*, Reproductive Health Research Unit, Johannesburg.

[CIT0013] Ehlers V.J., 2003, ‘Adolescent mothers’ knowledge and perceptions of contraceptives in Tshwane, South Africa’, *Health SA Gesondheid* 8(1), 13–25. 10.4102/hsag.v8i1.112

[CIT0014] Freedman M., 2013, *Birth control pills and weight gain*, viewed 14 October 2015, from http://www.healthgrades.com/explore/birth-control-pills-and-weight-gain.

[CIT0015] Govender P., 2010, ‘Pre-teens are having plenty of unsafe sex, shock survey finds’, *Sunday Times*, 22 August, p. 6.

[CIT0016] Grant M.J., & Hallman K.K., 2008, ‘Pregnancy-related school dropout and prior school performance in KwaZulu-Natal, South Africa’, *Studies in Family Planning* 39(4), 369–382. PMID: 19248721. 19248721 10.1111/j.1728-4465.2008.00181.x

[CIT0017] Holcomb M.A., Williams R. & Skipper M.T., 2009, ‘Adolescent contraception: Sorting out the facts’, *The Nurse Practitioner* 34(9), 18–26. PMID: 19713817, 10.1097/01.NPR.0000360144.05066.e6 19713817

[CIT0018] Ishikawa H. & Yano E., 2008, ‘Patient health literacy and participation in the health-care process’, *Health Expectations* 11(2), 113–122. PMID: 18494956, 10.1111/j.1369-7625.2008.00497.x 18494956 PMC5060442

[CIT0019] Kickbusch I.S., 2001, ‘Health literacy: Addressing the health and education divide’, *Health Promotion International* 16(3), 289–297. PMID: 11509466, 10.1093/heapro/16.3.289 11509466

[CIT0020] Lincoln Y.S. & Guba E.G., 1985, *Naturalistic inquiry*, Sage, Beverly Hills, CA.

[CIT0021] LoBiondo-Wood G. & Haber J., 2010, *Nursing research: Methods and critical appraisals for evidence-based practice**,* 6th edn., Mosby, St. Louis, MO.

[CIT0022] Maja T.M., 2007, ‘Involvement of males in promoting reproductive health’, *Curationis* 30(1), 71–76. PMID: 17515319. 17515319 10.4102/curationis.v30i1.1054

[CIT0023] Manganello J.A., 2008, ‘Health literacy and adolescents: A framework and agenda for future research’, *Health Education Research* 23(5), 840–847. PMID: 18024979, 10.1093/her/cym069 18024979

[CIT0024] Mangiaterra V., Pendse R., McClure K. & Rosen J., 2008, ‘Adolescent pregnancy’, *Making Pregnancy Safe Notes* 1(1), 1–4.

[CIT0025] Marino J.L., Skinner S.R., Doherty D.A., Rosenthal S.L., Cooper Robbins S.C., Cannon J. *et al.*, 2013, ‘Age at menarche and age at first sexual intercourse: A prospective cohort study’, *Pediatrics* 132(6), 1028–1036. PMID: 24218473, 10.1542/peds.2012-3634 24218473

[CIT0026] Mason M.J, Tanner J.F, Piacentini M., Freeman D., Anastasia T., Batat W. *et al.*, 2013, ‘Advancing a participatory approach for youth risk behavior: Foundations, distinctions, and research directions’, *Journal of Business Research* 66(8), 1235–1241. 10.1016/j.jbusres.2012.08.017

[CIT0027] Mataboge M.L.S., Beukes S. & Nolte A.G.W., 2014, ‘Low functional health literacy, misconceptions and risks regarding prevention of unintended pregnancy, STIs, HIV and AIDS’, *African Journal for Physical, Health Education, Recreation and Dance* 1(1), 127–139.

[CIT0028] Mchunu G., Peltzer K., Tutshana B. & Seutlwadi L., 2012, ‘Adolescent pregnancy and associated factors in South African youth’, *African Health Sciences* 12(4), 426–434. PMID: 23515418. 23515418 10.4314/ahs.v12i4.5 PMC3598281

[CIT0029] Mpofu M., 2012, ‘Teen pregnancies roil education authorities’, *Pretoria News*, 9 July, p. 1.

[CIT0030] Nelson L.E., Morrison-Beedy D., Kearney M.H. & Dozier A., 2011, ‘Always, never, or sometimes: Examining variation in condom-use decision making among black adolescent mothers’, *Research in Nursing* & *Health* 34(4), 270–281. PMID: 21633960, 10.1002/nur.20445 21633960 PMC3412046

[CIT0031] Peltzer K. & Chirinda W., 2014, ‘Access to opportunities and the lovelife programme among youth in South Africa’, *Journal of Psychology in Africa* 23(1), 77–84

[CIT0032] Polit D.F. & Beck C.T., 2010, *Essentials of nursing research: Appraising evidence for nursing practice*, 7th edn., Wolters Kluwer Health, Lippincott Williams & Wilkins, Philadelphia, PA.

[CIT0033] Polit D.F. & Beck C.T., 2012*,* *Nursing research: Generating and assessing evidence for nursing practice**,* 9th edn., Wolters Kluwer Health, Lippincott Williams & Wilkins, Philadelphia, PA.

[CIT0034] Ramkissoon A., Searle C., Burns C & Beksinska M.E., 2010, ‘Sexual and reproductive health and rights’, *South African Health Review* 2010, 33–47.

[CIT0035] South African Broadcasting Company [SABC], 2010–2011, [Advertisement], South African Television Services.

[CIT0036] South African Broadcasting Company [SABC] News, 2013, *SA school pregnancy reaches early primary school levels* –*report*, 3 October 2013, South African Television Services, viewed 4 October 2015, from http://www.sabc.co.za/news/a/a0ee2100414f5e638295873895839b19/SA-school-pregnancy-reaches-early-primary-school-levels—report.

[CIT0037] South African Broadcasting Company [SABC] News, 2015 *Education dept worried about 20,000 teenage pregnancies*, 25 March 2015, South African Television Services, viewed 9 October 2015, from http://ewn.co.za/2015/03/25/Education-dept-worried-about-20000-teenage-pregnancies.

[CIT0038] Streubert H.J. & Carpenter D.R., 2007*,* *Qualitative research in nursing: Advancing the humanistic imperative*, 4th edn., Wolters Kluwer Health, Lippincott Williams & Wilkins, Philadelphia, PA.

[CIT0039] Tesch R., 1990, *Qualitative Research: Analysis Types* & *Software Tools*, Falmer Press, Bristol, PA.

[CIT0040] UK Government, 2014, *Drug safety update. St John*’*s wort: Interaction with hormonal contraceptives, including implants*, viewed 14 October 2015, from https://www.gov.uk/drug-safety-update/st-john-s-wort-interaction-with-hormonal-contraceptives-including-implants.

[CIT0041] Willan S., 2013, *A review of teenage pregnancy in South Africa* – *Experiences of schooling, and knowledge and access to sexual* & *reproductive health services*, Partners in Sexual Health, Witwatersrand University.

[CIT0042] Wu L., 2010, ‘A survey on the knowledge, attitude, and behavior regarding contraception use among pregnant teenagers in Beijing, China’, *Clinical Nursing Research* 19(4), 403–415. PMID: 20656920, 10.1177/1054773810375982 20656920

[CIT0043] Xu J.S., Chen Y. & Cheng L.N., 2005, ‘A questionnaire survey of pregnant teenagers on knowledge and behavior of sexuality and contraception in Shanghai’, *Maternal and Child Health Care of China* 20, 1184−1186.

[CIT0044] Ziyane I.S. & Ehlers V.J., 2007, ‘Swazi men's contraceptive knowledge, attitudes, and practices’, *Journal of Transcultural Nursing* 18(1), 5–11. PMID: 17202523, 10.1177/1043659606294190 17202523

